# Genome rearrangements with duplications

**DOI:** 10.1186/1471-2105-11-S1-S27

**Published:** 2010-01-18

**Authors:** Martin Bader

**Affiliations:** 1 Institute of Theoretical Computer Science, Ulm University, 89069 Ulm, Germany

## Abstract

**Background:**

Finding sequences of evolutionary operations that transform one genome into another is a classical problem in comparative genomics. While most of the genome rearrangement algorithms assume that there is exactly one copy of each gene in both genomes, this does not reflect the biological reality very well - most of the studied genomes contain duplicated gene content, which has to be removed before applying those algorithms. However, dealing with unequal gene content is a very challenging task, and only few algorithms allow operations like duplications and deletions, especially if the duplicated or deleted segments are of arbitrary size.

**Results:**

We recently proposed a heuristic algorithm for sorting unichromosomal genomes by reversals, block interchanges, tandem duplications, and deletions. In this paper, we extend this approach to multichromosomal genomes. We are now able to sort a multichromosomal ancestral genome into a genome of a descendant by a large set of different operations, including tandem duplications and deletions of segments of arbitrary size.

**Conclusion:**

Experimental results show that our algorithm finds sorting sequences that have a weight close to the true evolutionary distance.

## Background

During evolution, genomes are subject to genome rearrangements, which are large scale mutations that can alter the ordering and orientation (strandedness) of the genes on the chromosomes or even change the genome content by inserting, deleting, or duplicating genes. Because these events are rare compared to point mutations, they can give us valuable information about ancient events in the evolutionary history of organisms. For this reason, one is interested in the most "plausible" genome rearrangement scenario between two genomes. More precisely, given two genomes, one wants to find an optimal sequence of rearrangements that transforms this genome into the other. In the classical approach, each gene has exactly one copy in each genome, and only operations that do not change the genome content are considered. A breakthrough in the research of these "classical operations" was Hannenhalli and Pevzner's algorithm for *sorting by reversals *[1], and due to further research, we are now able to sort multichromosomal genomes by *reversals, translocations, fusions, and fissions *in polynomial time [2,3]. If one also considers *transpositions*, the problem gets more complicated, and there are only approximation algorithms known [4-6]. To simplify the existing algorithms, Yancopoulos et al. invented the *double cut and join *operator (DCJ), which can simulate reversals, translocations, fusions, fissions, and block interchanges (a more generalized form of transpositions), resulting in a simple and efficient algorithm [7].

However, restricting the genes to be unique in each genome does not reflect the biological reality very well, as in most genomes that have been studied, there are some genes that are present in two or more copies. This holds especially for the genomes of plants, and one of the most prominent genomes is the one of the flowering plant *Arabidopsis thaliana*, where large segments of the genome have been duplicated (see e.g. [8]). There are various evolutionary events that can change the content of the genome, like duplications of single genes, horizontal gene transfer, or tandem duplications. For a nice overview in the context of comparative genomics, see [9]. In a pioneering work, Sankoff tackled the challenge of genomes with duplicated genes with his "exemplar model" [10], where the following problem was examined. Given two genomes with duplicated genes, identify in both genomes the "true exemplars" of each gene and remove all other genes, such that the rearrangement distance between these modified genomes is minimized. This approach was later extended to the "matching model", where one searches for a maximum matching between the copies of each gene such that the genome rearrangement distance according to this matching is minimized [11]. However, both approaches have been proven to be NP-hard for the breakpoint distance and the reversal distance [11-13]. Note that these approaches do not construct the evolutionary events that changed the genome contents, i.e. the set of operations is still restricted to the classical set of operations. The first approach that explicitly constructed duplication events was done by El-Mabrouk [14], where one searches for a hypothetical ancestor with unique gene content, such that the reversal and duplication distance from this ancestor to a given descendant (with duplicated genes) is minimized. This work has been further extended during the last years (see e.g. [13,15]). Still, the duplications were technically limited to have the length of one element, and therefore the algorithms can only be applied if no large segmental duplication happened during evolution. One idea to overcome this problem was to simulate duplications by insertions, as it has been done in [16-18]. Recently, Yancopoulos and Friedberg provided a mathematical model (but without algorithm) of a genome rearrangement distance for genomes with unequal gene content [19], combining the DCJ operator with arbitrary but length-weighted insertions and deletions. To the best of our knowledge, the first work that allowed duplications of arbitrary segments was done by Ozery-Flato and Shamir [20], who demonstrated that a simple greedy algorithm can find biologically realistic sorting scenarios for most karyotypes in the *Mitelman Database of Chromosome Abberations in Cancer *[21]. Further simplifications of the model led to an algorithm with provable approximation ratio of 3 [22] (note that the algorithm performs much better in practice). Recently, we proposed a heuristic algorithm for sorting a unichromosomal genome by DCJs, tandem duplications, and deletions of arbitrary segments [23]. In this work, we extend this approach to multichromosomal genomes. We are now able to sort an ancestral multichromosomal genome by a large set of operations, including duplications and deletions of arbitrary size. As a constraint, two chromosomes in the ancestral genome must be either disjoint or identical. Although this restriction seems to be very limiting, this is often fulfilled in practice. A possible application is to examine the evolution of a cancer karyotype out of a diploid set of healthy chromosomes.

## Methods

### Preliminaries

A *chromosome * is a string over the alphabet Σ = {1, ..., *n*}, where each element may have a positive or negative orientation (indicated by  or ). We get the *inverse *of an element  (indicated by ) by inverting its orientation. The *reflection *of a chromosome  is the chromosome . It is considered to be equivalent to *π*^*i*^. A *genome π*= {*π*^*i*^, ..., *π*^*m*^} is a multiset of chromosomes. The *multiplicity mult*(*π*, *x*) of an element *x *is the number of its occurrences (with arbitrary orientation) in *π*. A *segment *is a consecutive sequence of elements of a chromosome. Each element *x *can also be represented by the ordered set of its *extremities x*_*t*_(the *tail *) and *x*_*h*_(the *head *), where the ordering of the extremities is *x*_*t *_*x*_*h *_if *x *has positive orientation, and *x*_*h *_*x*_*t *_otherwise. For example, the chromosome  can also be written as (1_*t*_1_*h*_2_*t*_2_*h*_). The two extremities belonging to the same element are called *co-elements*. We say the tail/head *x*_*t/h *_of an element *x *is a *telomere *if there is a chromosome in *π *beginning or ending with *x*_*t */*h*_. The value *t*(*π*, *x*_*t*_) determines how often *x*_*t *_is a telomere in *π*, the value *t*(*π*, *x*_*h*_) is defined analogously. Two consecutive extremities in a chromosome *π*^*i *^which are no co-elements form an *inner adjacency *w.r.t. another genome *ρ *if they are also consecutive in a chromosome of *ρ*, otherwise they form an *inner breakpoint*. An extremity which is a telomere in *π *forms a *telomere adjacency *w.r.t. another genome *ρ *if it is also a telomere in *ρ*, otherwise it forms a *telomere breakpoint*. For example, if *ρ *= {(1_*t*_1_*h*_2_*t*_2_*h*_3_*t*_3_*h*_)} and *π *= {(1_*t*_1_*h*_2_*t*_2_*h*_), (1_*t*_1_*h*_3_*t*_3_*h*_)}, then 1_*t *_and 3_*h *_form telomere adjacencies, while 2_*h *_forms a telomere breakpoint. Applying an *operation op *to a genome *π *yields the genome *op*(*π*). A *genome rearrangement problem *is defined as follows. Given two genomes *ρ *and *π *and a set of possible operations, where each operation is assigned a weight, find a sequence of minimum weight (i.e. the sum of the weights of the operations is minimized) that transforms *ρ *into *π*. This minimum weight will be denoted by *d*(*ρ*, *π*). In our algorithm, we will consider all operations listed in Subsection "Operations". For simplification, we will assume that two chromosomes in *ρ *are either disjoint (i.e. they have no element in common) or identical. Furthermore, each element in Σ must appear in at least one chromosome of *ρ*. Note that these restrictions only hold for the genome *ρ*, not for *π*.

### Operations

The following set of operations will be considered by our algorithm. A *reversal *inverts the order of the elements of a segment. Additionally, the orientation of each element in the segment is inverted. A *transposition *cuts a segment out of a chromosome, and reinserts it at another position in the same chromosome. If we additionally apply a reversal on this segment, we speak of an *inverted transposition*. A *fusion *concatenates two chromosomes. Both chromosomes *π*^*i *^and *π*^*j *^can be inverted before the operation, i.e. we can replace *π*^*i *^or *π*^*j *^by its reflection. A *fission *splits a chromosome into two chromosomes. A *translocation *splits two chromosomes *π*^*i *^and *π*^*j *^into *π*^*i*, *t*^, *π*^*i*, *h *^and *π*^*j*, *t*^, *π*^*j*, *h*^, and then concatenates *π*^*i*, *t *^with *π*^*j*, *h *^and *π*^*j*, *t *^with *π*^*i*, *h*^. Again, both chromosomes can be inverted before the operation. A *tandem duplication *inserts an identical copy of a segment immediately after this segment in a chromosome. A *transposition duplication *inserts an identical copy of a segment at an arbitrary position in the genome. A *deletion *cuts a segment out of a chromosome. A *chromosome duplication *creates an identical copy of a chromosome. A *chromosome deletion *deletes a chromosome.

All operations have weight 1, except for (inverted) transpositions and transposition duplications, which have weight 2. These weights are chosen rather by mathematical than biological reasons, but still result in biologically realistic scenarios (see also Section "Discussion"). To simplify the analysis of the effects of the operations in Section "A lower bound", we will there use the *double cut and join operator *(DCJ), which has been introduced in [7]. A DCJ cuts a genome at two positions, and rejoins the cut ends in two new pairs. It is a well-known fact that reversals, translocations, fusions, and fission can be described by one DCJ operation, while transpositions can be described by two DCJ operations. Duplications and deletions cannot be described by DCJ operations and therefore must be examined separately.

### The breakpoint graph

**Figure 1:**
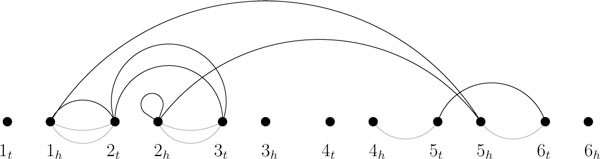
**The breakpoint graph**. The breakpoint graph of  and . Note that there is no black edge (4_*h*_, 3_*h*_), as 3_*h*_is a telomere in *ρ*. The edge (2_*t*_, 3_*t*_) has a multiplicity of 2, all other black edges have a multiplicity of 1. The edge (2_*h*_, 2_*h*_) is a loop. The breakpoint graph consists of 5 components, the black edge (5_*t*_, 6_*t*_) is a 1-bridge, the pair of black edges (1_*h*_, 5_*h*_), (2_*h*_, 5_*h*_) is a 2-bridge.

Our main tool for developing the algorithm is the breakpoint graph, which is an edge-colored multigraph that visualizes the current adjacencies and breakpoints. It has been introduced by Bafna and Pevzner to solve rearrangement problems on genomes without duplicated genes [24]. We extend this graph such that it can also be used for genomes with duplicated genes. The breakpoint graph of two genomes *ρ *and *π *can be constructed as follows. First, we write the set of vertices {1_*t*_, 1_*h*_, 2_*t*_, 2_*h*_, ..., *n*_*t*_, *n*_*h*_} from left to right on a straight line. Second, for each pair (*x*_*t/h*_, *y*_*t/h*_) of extremities that are no co-elements but consecutive in a chromosome of *ρ*, we connect the corresponding vertices by a *gray edge*. Third, we analogously add a *black edge *for each pair of extremities that are consecutive in *π*. However, if one of the endpoints is not an endpoint of a gray edge (i.e. it corresponds to a telomere in *ρ*), we do not add the black edge (this is required to obtain a good lower bound in Section "A lower bound"). For an example, see Fig. 1. In contrast to the original breakpoint graph, each vertex can be the endpoint of several gray and black edges. The *multiplicity *of an edge (*v*, *v'*) is the number of black edges between *v *and *v'*. A black edge (*v*, *v*) is called a *loop*. Let *L*(*ρ*, *π*) denote the number of vertices with a loop. Two vertices *v*, *v' *are in the same *component *of the graph if and only if there is a path (consisting of gray and black edges) from *v *to *v'*. Let *C*(*ρ*, *π*) denote the number of components in the breakpoint graph of *π*. A black edge is called a *1-bridge *if the removal of this edge increases *C*(*ρ*, *π*). A pair of black edges is called a *2-bridge *if none of the edges is a 1-bridge and the removal of both edges increases *C*(*ρ*, *π*).

### A lower bound

Instead of searching for a sequence of operations *op*_1_, ..., *op*_*k*_that sorts *ρ *into *π*, one can also search for the inverse sequence  that sorts *π *into *ρ *(we will call such a sequence a *sorting sequence*). This is more convenient, because then the breakpoint graph has some nice properties due to the limitations in *ρ*. Note that simply restricting *π *instead of *ρ *would not work, because the operations are directed from the restricted ancestral genome to the unrestricted descendant, i.e. we would nevertheless have to invert the operations. In the following, we will examine what effects the inverse operations have on the breakpoint graph. In the unichromosomal case, we were mainly interested in the effects on components and loops (see [23]). As the breakpoint graph does not contain edges for telomeres, we additionally have to consider the amount of *incorrect telomeres *(denoted by *T*(*ρ*, *π*)), which is defined as follows.

In other words, for each telomere *x*_*t*_(or *x*_*h*_) in *ρ*, we count how often we must create this telomere in *π *such that *t*(*ρ*, *x*_*t*_) ≤ *t*(*π*, *x*_*t*_) (or *t*(*ρ*, *x*_*h*_) ≤ *t*(*π*, *x*_*h*_)). Additionally, we add the amount of telomeres *x*_*t *_and *x*_*h *_in *π *that have to be removed, i.e. they are not telomeres in *ρ*.

**Lemma 1**. *If π *= *ρ*, *then C*(*ρ*, *π*) *is maximized*, *and L*(*ρ*, *π*) *and T*(*ρ*, *π*) *are minimized*.

*Proof*. If *π *= *ρ*, the set of gray edges and the set of black edges in the breakpoint graph are equal. Thus, removing black edges does not increase the number of components, and adding black edges can only decrease the number of components. Therefore, changing *π *cannot increase *C*(*ρ*, *π*). *L*(*ρ*, *π*) and *T*(, *π*) are both positive numbers, and if *π *= *ρ*, they are equal to 0 and therefore minimized.   □

In [23], we have shown that all operations can either change the number of components by 1, or change the number of loops by 2. These observations still hold for our slightly modified breakpoint graph. We will now examine how an operation can change *T*(*ρ*, *π*).

### Inverse reversal, translocation, transposition

These operations can be simulated by one or two inverse DCJs (which is equivalent to a normal DCJ), thus it is sufficient to examine the effects of a DCJ (note that transpositions, which require two DCJs, have weight 2). A DCJ can only change *T*(*ρ*, *π*) if one of its edges is the end of a chromosome. Then, a telomere *x*_*t */*h *_is removed and a new telomere *y*_*t */*h *_is created. This decreases *T *(*ρ*, *π*) by 1 or 2 if *x*_*t */*h *_is not a telomere in *π *and *y*_*t */*h *_is a telomere in *π*, otherwise the operation does not decrease *T*(*ρ*, *π*). However, in the first case, the DCJ did not cut any black edge, as we neither draw black edges for telomeres in *ρ *nor for telomeres in *π*.

### Inverse fusion (fission)

Splitting a chromosome will only decrease *T*(*ρ*, *π*) if both new telomeres are also telomeres in *ρ*. In this case, no black edge in the breakpoint graph is removed, i.e. *C*(*ρ*, *π*) and *L*(*ρ*, *π*) remain unchanged. *T*(*ρ*, *π*) is decreased by at most 2.

### Inverse fission (fusion)

Concatenating two chromosomes can decrease *T*(*ρ*, *π*) by at most 2. This operation never removes a black edge, thus *C*(*ρ*, *π*) cannot be increased and *L*(*ρ*, *π*) cannot be decreased.

### Inverse tandem duplication

This operation does never change the set of telomeres in *π*, and therefore cannot change *T*(*ρ*, *π*).

### Inverse transposition duplication

This operation can decrease *T*(*ρ*, *π*) only if the duplicated segment is at a chromosome end, and the new chromosome end (after deleting the segment) is a telomere in *ρ*. In this case, no black edge with multiplicity 1 is removed, therefore *C*(*ρ*, *π*) and *L*(*ρ*, *π*) remain unchanged. The decrement of *T*(*ρ*, *π*) is ≤ 2.

### Inverse deletion (insertion)

This operation can only change *T*(*ρ*, *π*) if we insert a segment at a chromosome end. In this case, no black edge is removed, i.e. *C*(*ρ*, *π*) cannot be increased and *L*(*ρ*, *π*) cannot be decreased. *T*(*ρ*, *π*) is decreased by at most 2.

### Inverse chromosome duplication

This operation can decrease *T*(*ρ*, *π*) by at most 2 (if the telomeres of the removed chromosome are not telomeres in *ρ*). Only black edges with multiplicity ≥ 2 are removed, thus *C*(*ρ*, *π*) and *L*(*ρ*, *π*) remain unchanged.

### Inverse chromosome deletion (chromosome insertion)

This operation can decrease *T*(*ρ*, *π*) by at most 2 (if the telomeres of the new chromosome are also telomeres in *ρ*). In the breakpoint graph, no black edges are removed, i.e. *C*(*ρ*, *π*) cannot be increased and *L*(*ρ*, *π*) cannot be decreased.

**Theorem 1**. *A lower bound lb*(*ρ*, *π*) *of the distance d*(*ρ*, *π*) *is*

*where c*(*ρ*) = *n *+ (*number of different chromosomes in ρ*), *and L_i_*(*ρ*, *π*) *is the number of vertices with a loop in component C*_*i *_*in the breakpoint graph of ρ and π*.

*Proof*. An operation that decreases *T*(*ρ*, *π*) will neither increase *C*(*ρ*, *π*) nor decrease *L*(*ρ*, *π*), therefore we can separate every sequence into operations that decrease *T*(*ρ*, *π*) and operations that decrease . Each operation decreases *T*(*ρ*, *π*) by at most 2, so we need at least  operations of the first kind. Furthermore, if *ρ *= *π*, then *C*(*ρ*, *π*) = *c*(*ρ*) and therefore *CL*(*ρ*, *π*) = 0. As each operation decreases *CL*(*ρ*, *π*) by at most one (the proof in [23] still holds), we need at least *CL*(*ρ*, *π*) operations of the second kind. Therefore, any sorting sequence must have at least *lb*(*ρ*, *π*) operations.   □

**Corollary 1**. *lb*(*ρ*, *ρ*) = 0.

Unfortunately, there are genomes *π *≠ *ρ *with *lb*(*ρ*, *π*) = 0, i.e. it is not sufficient to sort until the lower bound reaches 0. We therefore have to introduce another distance measure *τ*(*ρ*, *π*). We will use the following definitions.

For example, if *ρ *=  and *π *= , then *lb*(*ρ*, *π*) = 0 and *τ*(*ρ*, *π*) = 2.

**Lemma 2**. *If ρ *= *π*, *then τ*(*ρ*, *π*) = 0. *Otherwise*, *τ*(*ρ*, *π*) > 0.

*Proof*. It is clear that *τ*(*ρ*, *π*) = 0 if *ρ *= *π*. In order to minimize *τ*(*ρ*, *π*), it is necessary to minimize *m*(*ρ*, *π*) and to maximize *ia*(*ρ*, *π*) and *ta*(*ρ*, *π*). *ia*(*ρ*, *ρ*) and *ta*(*ρ*, *ρ*) are independent of *π *and therefore fixed. Each inner adjacency is weighted by 2. We can interpret this as if each of the adjacent extremities is weighted by 1. Therefore, we can say that each element in *π *can account at most 2 to *ia*(*ρ*, *π*) + *ta*(*ρ*, *π*), and this value is reached if there is an adjacency on both sides of the element. Thus, the contribution to *τ*(*ρ*, *π*) of all occurrences of an element *x *in *π *is minimized if *mult*(*ρ*, *x*) = *mult*(*π*, *x*) and no extremity of *x *is part of any breakpoint. Every additional occurrence of *x *may increase *ia*(*ρ*, *π*) + *ta*(*ρ*, *π*) by 2, but also increases *m*(*ρ*, *π*) by 4 and therefore increases *τ*(*ρ*, *π*) by at least 2. This means that *τ*(*ρ*, *π*) is minimized if each element has the same multiplicity in *ρ *and *π*, and the breakpoint graph contains no breakpoints. This is the case if and only if *ρ *and *π *are identical.   □

### The algorithm

The algorithm uses a greedy strategy to sort *π *into *ρ *by inverse operations. For better readability, we will simply write "operation" instead of "inverse operation" in this section. First, we define Δ*lb*(*op*) = *lb*(*ρ*, *π*) - *lb*(*ρ*, *op*(*π*)) and Δ*τ*(*op*) = *τ*(*ρ*, *π*) - *τ*(*ρ*, *op*(*π*)). The *score σ *of an operation *op *is defined as the tuple *σ*(*op*) = (Δ*lb*(*op*), Δ*τ*(*op*)). The comparison operator between two scores is defined by *σ*(*op*_1_) >*σ*(*op*_2_) if Δ*lb*(*op*_1_) > Δ*lb*(*op*_2_) ∨ Δ(*lb*(*op*_1_) = Δ*lb*(*op*_2_) ∧ Δ*τ *(*op*_1_) > Δ*τ *(*op*_2_)). In each step, we search for operations that decrease the lower bound, and apply the one with the greatest score. If no such operation exists, we use additional heuristics to find operations that do not change the lower bound but have a positive score (i.e. *σ *(*op*) >(0, 0)). There is still the possibility that we even do not find such an operation. In this case, we use a fallback algorithm that is guaranteed to terminate.

#### Operations that decrease the lower bound

Finding operations that increase *C*(*ρ*, *π*) can be done by finding 1-bridges and 2-bridges in the breakpoint graph and verifying additional preconditions, as shown in [23]. The only difference is that now a DCJ can cut only one black edge. This is the case when the other cutting point contains a telomere in *ρ *or *π*. Thus, we also have to consider DCJs that act on a 1-bridge and a telomere. Such a DCJ can be interpreted as inverse reversal, translocation, fission, or transposition. In the last case, we have to find a third cutting point in the same chromosome such that the resulting inverse transposition still increases *C*(*ρ*, *π*). Also finding operations that decrease *L*(*ρ*, *π*) is straightforward and can be done as in [23]. The remaining task is to find operations that decrease *T*(*ρ*, *π*). For this, we create a list of telomeres in *π *that are not telomeres in *ρ*, and another list of inner breakpoints in *π *where at least one of the adjacent elements is a telomere in *ρ*. Operations that decrease *T*(*ρ*, *π*) must act on one or two points of these lists, depending on the operation type. Creating the lists can be done by a linear scan over *π*, therefore all operations that decrease *T*(*ρ*, *π*) can be found in quadratic time. The only exceptions are inverse deletions and inverse chromosome deletions, which may add segments of arbitrary content. Practical tests have shown that it is better to only allow the insertion of segments without any breakpoints. This does not only lead to better sorting sequences, but also keeps the time complexity of finding the operations in *O*(*n*^2^).

#### Additional operations

If there is no operation that decreases *lb*(*ρ*, *π*), we may still find operations that do not change the lower bound but decrease *τ*(*ρ*, *π*). Searching for all these operations would exceed our computing capacity, so we just search for the following subset of these operations that can be found easily.

• There are inverse tandem duplications and transposition duplications that do not change *σ*(*ρ*, *π*), but decrement *τ*(*ρ*, *π*). We therefore search for identical consecutive segments that are maximal, i.e. they cannot be extended in any direction, and check the effect on *σ*(*ρ*, *π*) and *τ*(*ρ*, *π*) if we remove one of them. This operation corresponds either to an inverse tandem duplication, or to an inverse transposition duplication.

• Depending on the telomeres of a chromosome, the lower bound can remain unchanged during an inverse chromosome duplication, but *τ*(*ρ*, *π*) can decrease. We therefore search for identical chromosomes and check the score of removing one of them.

• Inserting a segment of consecutive elements *x *with *mult*(*ρ*, *x*) >*mult*(*π*, *x*) decreases *τ*(*ρ*, *π*). We search for such segments of maximal length and try to insert them by an inverse deletion. Note that this is not always possible as this operation can increase the lower bound by merging two components.

• Creating inner or telomere adjacencies never increases the lower bound, but decreases *τ*(*ρ*, *π*). We therefore search for DCJs and inverse fissions that create new adjacencies without splitting old ones.

#### The fallback algorithm

It is possible that there is neither an operation that decreases *lb*(*ρ*, *π*), nor an operation that decreases *τ*(*ρ*, *π*), so the main algorithm gets stuck. However, this case cannot occur if all elements have the same multiplicity in *ρ *and in *π*.

**Lemma 3**. *If ρ *≠ *π and mult*(*ρ*, *x*) = *mult*(*π*, *x*) *holds for all elements x*, *then there is an operation with positive score*.

*Proof*. When the preconditions are fulfilled, there must be at least one breakpoint in *π*. We have to distinguish three cases. (1) This is a telomere breakpoint. W.l.o.g. a chromosome in *π *ends with *x*_*h*_, but *x*_*h *_is not a telomere in *ρ*. Then, *mult*(*ρ*, *x*) = *mult*(*ρ*, *x *+ 1) (as they are in the same chromosome), and therefore there must be another breakpoint including (*x *+ 1)_*t*_. An operation that creates an adjacency between *x*_*h *_and (*x *+ 1)_*t *_will not decrease the lower bound, but decrease *τ *(*ρ*, *π*) by at least 2. (2) The breakpoint is an inner breakpoint between two extremities that are telomeres in *ρ*. In this case, the score of cutting the chromosome at this breakpoint is (1, 2), because both extremities become telomeres (i.e. *T*(*ρ*, *π*) increases by 2), and we create two telomere adjacencies. (3) The breakpoint is an inner breakpoint, and at least one of the adjacent extremities is not a telomere in *ρ*. W.l.o.g., the breakpoint is of the form (*x*_*h*_, *y*_*h*_), and *x*_*h *_is not a telomere in *ρ*. Then, *mult*(*ρ*, *x*) = *mult*(*ρ*, *x *+ 1), thus there must be another breakpoint including (*x *+ 1)_*t*_. An operation that creates an adjacency between *x*_*h *_and (*x *+ 1)_*t *_will not increase the lower bound, but decrease *τ*(*ρ*, *π*) by at least 2.   □

The fallback algorithm will first ensure that the precondition of the lemma holds. For each chromosome *ρ*^*i *^in *ρ*, we determine the element *x *with the most occurrences in *π*. We then create maximal segments of consecutive elements  ... such that each element *z *in the segment belongs to *ρ*^*i *^and *mult*(*π*, *z*) <*mult*(*π*, *x*), and add this segment by an inverse deletion to *π*. Note that this can be done without creating new breakpoints. This step is repeated until all elements belonging to the same component in *ρ *have the same multiplicity in *π*. We then transform *ρ *into *ρ' *by applying chromosome duplications and chromosome deletions on *ρ *such that for each element *x*, *mult*(*ρ'*, *x*) = *mult*(*π*, *x*). Now, we apply our normal algorithm to sort *π *into *ρ'*. To ensure that the precondition of Lemma 3 is always fulfilled, we forbid operations that create or delete elements, i.e. any kind of duplication or deletion. Due to Lemma 3, the algorithm will find a sorting sequence that transforms *π *into *ρ'*. As last step, we have to undo the operations that transformed *ρ *into *ρ'*.

## Results

We tested our algorithm on artificial data and on cancer karyotypes from the "Mitelman Database of Chromosome Aberrations in Cancer" [21].

### Artificial data

**Figure 2:**
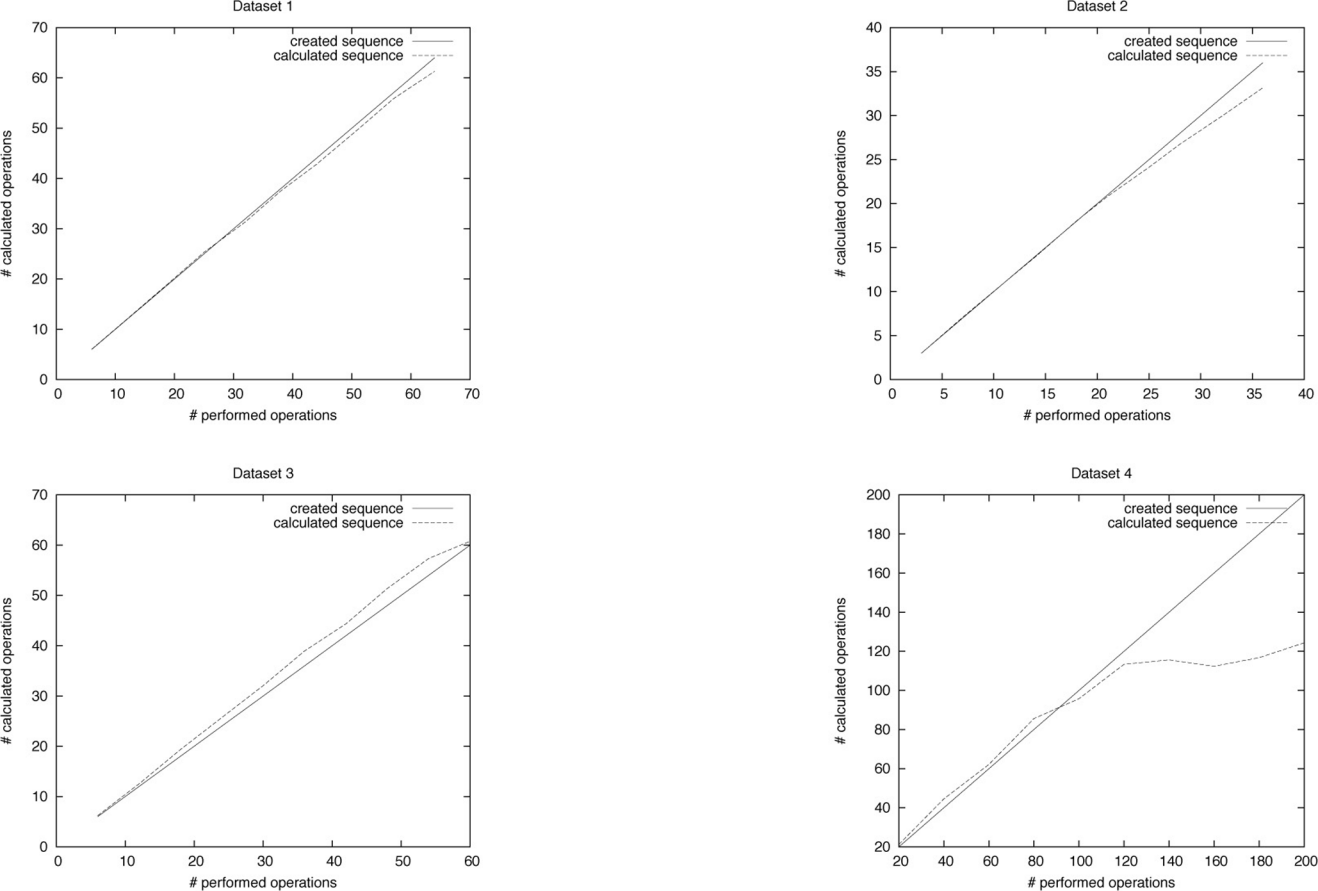
**Results on artificial data**. The relation of the created distance, the calculated distance, and the lower bound for the different artificial datasets.

We used simulated data to assess the performance of our algorithm. First, we created genomes *ρ *with *n *different elements and *c *different chromosomes. Each chromosome has the same size, the ploidy (i.e. the number of identical copies) of the chromosomes is 1 or 2. Then, we generated the genome *π *by applying random sequences of operations of weight *w *= *αn *on *ρ *(with *α *varying from 0.1 to 1.0 in steps of 0.1). The operations of the sequences are independently distributed, with all operations having the same probability. Although these probabilities do not match the biological reality, this is still convenient to assess the performance of the algorithm. Once the type of an operation was determined, the operation was drawn from a uniform distribution of all operations of this type. The genomes *ρ *and *π *were now used as input to our algorithm. The parameters *n *and *c *were chosen such that they reflect the properties of biologically meaningful datasets. To understand what "biologically meaningful" means, let us have a brief look on biological datasets. In most of them, elements do not represent single genes but *synteny blocks*, i.e. regions of a chromosome that are highly conserved and do not contain breakpoints. These synteny blocks normally contain several genes. The amount *n *of different synteny blocks depends on the allowed dissimilarity between the synteny blocks as well as on the evolutionary distance between the genomes. For example, El-Mabrouk et al. [25] tested their algorithm on yeast genomes with 55 synteny blocks, Zheng et al. [26] identified 34 synteny blocks between rice, sorghum, and maize. Salse et al. [27] used 60 synteny blocks to compare *Arabidopsis thaliana *and rice. A recent comprehensive study of *Drosophila *genomes [28] identified between 112 and 1406 synteny blocks, depending on the evolutionary distance of the species. Our datasets reflect those parameters. Dataset 1 contains 16 chromosomes of ploidy 2 with a total of 64 elements, this approximately matches the yeast genome. Dataset 2 contains 12 chromosomes of ploidy 2 with a total of 36 elements, Dataset 3 contains 5 chromosomes of ploidy 2 with a total of 60 elements. These are realistic values for plant genomes. Dataset 4 contains 5 different chromosomes with a total of 200 elements, two of them with ploidy 1 and three of them with ploidy 2 (corresponding to 2 sex chromosomes and 3 diploid chromosomes). This reflects the values for closely related *Drosophila *species. Each dataset contains 100 different test cases for each generated distance *w*. Together with the use of different distances *w*, this allows us to get a much more robust result than just testing on a few biological datasets. The results of our experiments are depicted in Fig. 2. The diagrams show that, on average, the calculated distance and the true evolutionary distance *w *lie close together. In many cases, the calculated scenarios were even slightly shorter than the true distance. In the fourth diagram, an additional saturation effect can be observed, i.e. we can find a sorting sequence with weight ≲130 for most genomes *π*, independent of the true distance *w*.

### Cancer karyotypes

**Figure 3:**
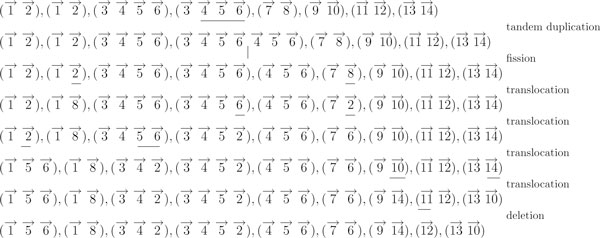
**An example sorting scenario**. Sorting scenario of a cancer karyotype that was reported in [30]. For better readability, all chromosomes that are identical in *ρ *and *π *are removed.

The "Mitelman Database of Chromosome Aberrations in Cancer" [21] contains the descriptions of cancer karyotypes which have been manually collected from publications over the last twenty years. For our experiments, we used the version of May 14, 2009, which contains 56428 datasets. The data is represented in the ISCN format, which can be parsed by the software tool CyDAS[29]. From all datasets which could be parsed by CyDAS without error (44064 datasets), we removed all unknown elements and compressed all segments without breakpoint, i.e. if a set of consecutive elements contains no breakpoint in any chromosome, it can be represented as one element. The resulting datasets were used as input to our algorithm. Most of the scenarios are rather easy to reconstruct, the average lower bound is 2.72 and the average calculated weight is 4.08. However, there are some more complicated karyotypes, with rearrangement scenarios of over 50 operations. Exemplarily, the reconstructed scenario for one karyotype is shown in Fig. 3. This karyotype was reported in [30], and can be described by the ISCN formula (for details about the ISCN format, see [31]).

In principle, our algorithm correctly identified all operations. The triple translocation *t*(3; 7; 9)(*q*23; *q*32; *q*22) and the new chromosome +*i*(7)(*q*10) are not allowed operations in our model. Our algorithm replaced the triple translocation by two translocations, and the new chromosome by a tandem duplication with a subsequent fission, which are the best possible explanations within our model.

## Discussion

In the last sections, we have shown that our algorithm will terminate in any case, and finds rearrangement scenarios of reasonable quality. However, the weights of the operations are chosen due to a mathematical model and do not reflect the biological reality. This leaves room for further investigations. For example, the algorithm could be improved by giving unwanted operations a larger weight or completely omit them. While adapting the theoretical model to other weights seems to be the obvious way to improve the algorithm, it might also be worth to examine how robust the results are w.r.t. the chosen weights. In other words, does the optimal rearrangement scenario change when we use other weights? Some observations in the genome can be explained at best by a specific operation (e.g. a duplicated chromosome is most likely caused by a single chromosomal duplication), no matter how this operation is weighted. Such observations are predominant in closely related genomes, and the corresponding operations can be reconstructed even with a wrong weighting scheme. In more diverged genomes, there are often different possible rearrangement scenarios, and the weighting scheme matters. Thus, further investigations should examine what the "critical distance" between two genomes is, i.e. up to which distance the optimal rearrangement scenario is mostly robust w.r.t. the weighting scheme.

## Conclusion

We presented an algorithm to sort multichromosomal genomes by a wide range of different operations. Although our results are promising, this algorithm should be seen as a single step towards an algorithm that produces biologically reliable results. While one direction of further research should investigate the chosen weighting scheme (see Section "Discussion"), other possible improvements are closer lower bounds or better heuristics.

## Competing interests

The authors declare that they have no competing interests.

## Authors' contributions

MB designed the algorithm, implemented it, performed the tests, and drafted this manuscript.
